# Discovery of Glycation Products: Unraveling the Unknown Glycation Space Using a Mass Spectral Library from In Vitro Model Systems

**DOI:** 10.1021/acs.analchem.3c05540

**Published:** 2024-02-12

**Authors:** Yingfei Yan, Daniel Hemmler, Philippe Schmitt-Kopplin

**Affiliations:** † Research Unit Analytical BioGeoChemistry, Helmholtz Zentrum München, Neuherberg 85764, Germany; ‡ Chair of Analytical Food Chemistry, Technical University of Munich, Freising 85354, Germany

## Abstract

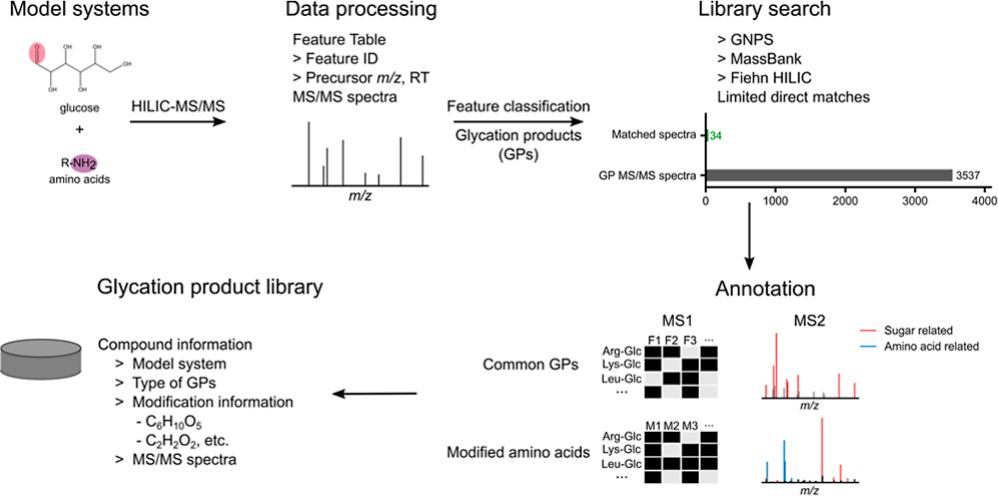

The nonenzymatic reaction between amino acids (AAs) and reducing sugars, also known as the Maillard reaction, is the primary source of free glycation products (GPs) in vivo and in vitro. The limited number of MS/MS records for GPs in public libraries hinders the annotation and investigation of nonenzymatic glycation. To address this issue, we present a mass spectral library containing the experimental MS/MS spectra of diverse GPs from model systems. Based on the conceptional reaction processes and structural characteristics of products, we classified GPs into common GPs (CGPs) and modified AAs (MAAs). A workflow for annotating GPs was established based on the structural and fragmentation patterns of each GP type. The final spectral library contains 157 CGPs, 499 MAAs, and 2426 GP spectra with synthetic model system information, retention time, precursor *m*/*z*, MS/MS, and annotations. As a proof-of-concept, we demonstrated the use of the library for screening GPs in unidentified spectra of human plasma and urine. The AAs with the C_6_H_10_O_5_ modification, fructosylation from Amadori rearrangement, were the most found GPs. With the help of the model system, we confirmed the existence of C_6_H_10_O_5_-modified Valine in human plasma by matching both retention time, MS1, and MS/MS without reference standards. In summary, our GP library can serve as an online resource to quickly screen possible GPs in an untargeted metabolomics workflow, furthermore with the model system as a practical synthesis method to confirm their identity.

## Introduction

Maillard reaction (MR) is well-known for contributing to flavors and colors during heat treatment in food processing.^1^ This reaction begins with forming Amadori rearrangement products (ARPs) through the condensation reaction between the carbonyl group of reducing sugars and the amine group of amino compounds.^2^ ARPs can further break down to produce highly reactive intermediates, such as aldehydes and α-dicarbonyls, which are continuously involved in the reaction and lead to diverse advanced glycation end products (AGEs). With the discovery of glycated hemoglobin in diabetic patients,^3^ MR has also been proven to occur under physiological conditions, resulting in free and protein-bound glycation products (GPs).^4^ The accumulation of endogenous GPs is associated with abnormal pathological conditions, like oxidative stress, diabetes, and chronic renal diseases.^5^

Liquid chromatography–mass spectrometry (LC–MS) is often used for GP analysis due to its high selectivity, sensitivity, and throughput. AGEs derived from lysine and arginine, including carboxymethyl-lysine (CML), carboxyethyl-lysine (CEL), glyoxal-hydroimidazolone (G-H), and methylglyoxal-hydroimidazolone (MG-H), are the most widely studied and targeted quantified GPs in food and biological samples because they can be produced by the reaction between glyoxal and methylglyoxal, two prevalent and reactive α-dicarbonyls, and nucleophilic side chains of lysine and arginine. In untargeted metabolomics studies, unknown GPs have been identified from either consumption of Maillard-modified food or endogenous formation. ARPs of lysine and leucylisoleucine were found in the feces of infants fed with formula due to MR during infant formula production and storage.^6^ The accumulation of ARPs of phenylalanine, methionine, lysine, proline, and citrulline was observed in the body fluid of patients with inborn errors in amino acid (AA) metabolism.^7^ Glycerate-modified AAs caused by highly reactive glycolytic intermediates were identified in the PARK7 knockout mouse brain.^8^ It is worth mentioning that the identification of GPs in the aforementioned studies was confirmed by chemically synthesized standards, which is time-consuming. Additionally, to the best of our knowledge, ARP MS/MS spectra of all 20 proteinogenic AAs are not available in public databases yet, despite that ARPs have been identified since 1937.^9^ In general, public databases suffer from available chemical structures and the MS/MS data of GPs.

The lack of MS/MS records in public libraries makes the annotation of GPs in untargeted metabolomics studies challenging. One common approach for obtaining MS/MS for compounds is to use standards. However, the limited number of commercially available GP standards compared with plentiful GPs makes this approach difficult to practice. Another strategy is in silico MS/MS generation based on compound structures by cheminformatics. This approach requires molecular structure templates to generate all possible structures,^10^ which is less suitable for compounds with high structural diversity like GPs. The model system is an ideal resource for producing a multitude of GPs in a reproducible way.^11,12^ Besides, the MR reaction between AAs and reducing sugars follows repeated reaction patterns.^13^ Typical reactions, such as sugar-amine condensation, dehydration series, redox reaction, and so forth, are observed in the reaction cascades and produce GPs with structural similarity across different model systems,^11,12^ which assists the annotation of GPs.

In this study, we aim to establish a GP spectral library for rapid GP screening. To extend the coverage of GPs, we analyzed model systems produced by the reaction of 20 proteinogenic AAs with glucose (Glc). Furthermore, we established a workflow to annotate GPs based on their structural characteristics derived from the MR pathway and fragmentation patterns of standards. Additionally, we demonstrated the utility of the spectral library together with model systems for identifying unknown GPs without reference standards.

## Experimental Section

### Materials and Reagents

Twenty AAs and 13 GPs purchased from different vendors were summarized in Table S1. The standard mixture of AAs and GPs at a concentration of 1 ppm was prepared in 80% acetonitrile (ACN). Ammonium formate (10 M stock solution) was obtained from Sigma-Aldrich (Steinheim, Germany). Formic acid (98% for mass spectrometry) was purchased from Honeywell Fluka (Charlotte, NC, USA). LC–MS grade ACN was purchased from Merck (Darmstadt, Germany). Pure water (18.2 MΩ) was produced by an in-house Milli-Q integral water purification system (Billerica, MA, USA). ESI-L low-concentration tuning mix was purchased from Agilent (Santa Clara, CA, USA). Lyophilized human plasma (P9523) was purchased from Sigma-Aldrich (St. Louis, MO, USA).

### Preparation of Maillard Model Systems

To prepare the model systems, 20 proteinogenic AA stock solutions (0.2 M) were mixed separately with a Glc stock solution (0.8 M) at a 1:1 (*v/v*) ratio in Milli-Q water. 0.1 M AA and 0.4 M Glc solutions were also prepared as control samples. For AAs with low solubility, including l-aspartic acid, l-glutamic acid, l-tryptophan, and l-tyrosine, standards were weighed individually in the vial, and then, the exact amount of 0.4 M Glc solution was added to make the concentration of AAs as 0.1 M. All solutions, including the control samples and model systems, were heated at 100 °C for 2 and 16 h, with triplicates prepared for each sample type.

### LC–MS/MS Analysis

Model systems were diluted 1:500 (*v/v*) by using 80% ACN for LC–MS/MS analysis. Lyophilized human plasma was first reconstituted with water and then extracted using ethanol with triplicates as previously described.^14−16^ For LC–MS/MS measurement, plasma ethanol extracts were evaporated and reconstituted in 80% ACN to achieve a 5-fold increase in concentration. Samples were analyzed using an ultrahigh-performance liquid chromatography system (Acquity, Waters, Milford, MA, USA) coupled with a quadrupole time-of-flight mass spectrometer (maXis, Bruker Daltonics, Bremen, Germany) using the method previously described.^17^ Briefly, a hydrophilic interaction chromatography ZIC-cHILIC (100 × 2.1 mm, 3 μm, 100 Å, zwitterionic, Merck, Darmstadt, Germany) column was used for LC separation. Eluent A was 5% ACN and eluent B was 95% ACN, with both 5 mM ammonium formate and 0.1% formic acid. The gradient was: 0 min, 99.9% B; 2 min, 99.9% B; 13 min, 56% B; 14 min, 30% B; 14.1 min, 10% B; 16 min, 10% B; 16.1 min, 99.9% B. The column was equilibrated under the initial condition for 3 min before each measurement. Injection volumes were 5 μL for model systems and the standard mixture and 10 μL for the plasma sample.

The diluted ESI-L low concentration tuning mix [1:4 (*v/v*) with 75% ACN] was injected in the first 0.3 min of every measurement for the internal mass spectrum calibration. The ESI source settings were as follows: nebulizer pressure 2 bar, dry gas flow 10 L/min, dry gas temperature 200 °C, and capillary voltage 4500 V. Mass spectra were recorded in positive electrospray mode with mass range of 50–1500 *m*/*z* and scan rate of 5 Hz. Data-dependent MS/MS experiments were performed after each MS scan to acquire MS/MS of the three highest MS1 ions with a collision energy of 25 eV.

### Data Processing

Raw LC–MS/MS data were calibrated and converted into an mzXML file using Bruker DataAnalysis (version 5.0). Each model system, along with its respective controls, including heated AAs and Glc, was processed separately as a group. Feature generation and corresponding MS/MS spectra extraction were achieved by XCMS (version 3.20.0).^18^ Feature grouping, isotope detection, and adduct annotation were performed by CAMERA (version 1.56.0).^19^ Detailed parameters for XCMS and CAMERA are listed in Table S2. In-source fragment (ISF) detection was based on the ISFrag package (version 0.1.0).^20^ Feature table cleaning, including removing signal with intensity less than 1500, features in solvent blank samples, isotope peaks, ISFs, and adducts except [M + H]^+^, was implemented by an in-house script in R.

Consensus MS/MS spectra of each feature were calculated by MSnbase package (version 2.24.2).^21^ MS/MS ions were aligned with *m*/*z* tolerance of 0.005 Da, and only peaks present in more than 25% of spectra were retained.^22^ The peaks with intensity less than 300 (roughly s/n of 3), and *m*/*z* larger than precursor *m*/*z* + 0.5 in each consensus spectrum were further removed to exclude noise and contaminant peaks. Spectra with peaks of less than 3 were removed. The cleaning of MS/MS spectra was supported by Spectra (version 1.8.3).^23^

To generate structural information in a homogeneous way, SIRIUS was used for formula calculation based on the MS/MS fragmentation tree.^24^ The annotations with correct elements (CHNO or CHNOS for cysteine and methionine) and exact precursor ion mass error less than 10 ppm were kept. GP spectra were identified by searching against publicly available databases, including GNPS,^25^ MassBank,^26^ and Fiehn HILIC.^27^ Three public MS/MS libraries were downloaded from MassBank of North America (https://massbank.us/downloads) in April 2023. The cosine spectral similarity score was calculated with OrgMassSpecR (Version 0.5-3).^28,29^ The annotation with precursor *m*/*z* difference <0.005 Da, cosine similarity >0.7, and matched fragment number with 0.01 Da tolerance ≥6 were kept. For the spectrum with multiple matches, only the annotation with the highest cosine score was retained.

### Chemical Pools Classification

Features were classified into three chemical pools based on the conception of Yaylayan.^30^ GPs, carbohydrate degradation products (CDPs), and AA degradation products (AADPs). Features exclusively detected in all three replicates of model systems but absent in the corresponding control samples were classified as GPs. Features also present in the heated-Glc and heated-AA control samples were categorized as CDPs and AADPs, respectively. Additionally, a few features were observed in both heated-Glc and heated-AAs controls, suggesting the possibility of the same compounds resulting from the degradation of both AAs and Glc, such as carboxylic acids.

### Repository Mining

The Unimod database was downloaded as XML format (https://www.unimod.org/downloads.html) in September 2023 and parsed to extract modification names, masses, and compositions. The modification list from Unimod was further filtered to keep only the masses within the modification mass range of modified AAs (MAAs) detected in our GP library. The modifications related to AA substitution were also removed because of their rare existence of model system reactions. For each MAA, its modification mass was searched against the filtered Unimod database with a mass tolerance of 0.005 Da for potential explanations.

Annotated recurrent unidentified spectra (ARUS) libraries containing spectra of unknowns in human plasma and urine were downloaded from the National Institute of Standards and Technology (NIST) Mass Spectrometry Data Center (https://chemdata.nist.gov/dokuwiki/doku.php?id=chemdata:arus).^14,31^ The match between the GP library and ARUS was conducted similarly to the spectral library search described in Data Processing.

The spectra of C_6_H_10_O_5_–MAAs of 20 proteinogenic AAs were searched against public data in GNPS/MassIVE repository through MASST.^32^ Both input and library spectra were filtered by removing fragments within ±17 Da of the precursor *m*/*z*. The mass tolerance of precursor ion and fragment ion was set as 0.005 and 0.01 Da, respectively. To achieve a low false discovery rate (<1%), a score above 0.7 and at least six matched fragments were required for matches of spectra.^33^ The species of the data set with matched spectra were assigned manually to eight categories, including food, environment, human, microbes, mouse, plant, rat, and others, according to data set organisms.

## Results and Discussion

### GP Profile in Model Systems

**Figure 1:**
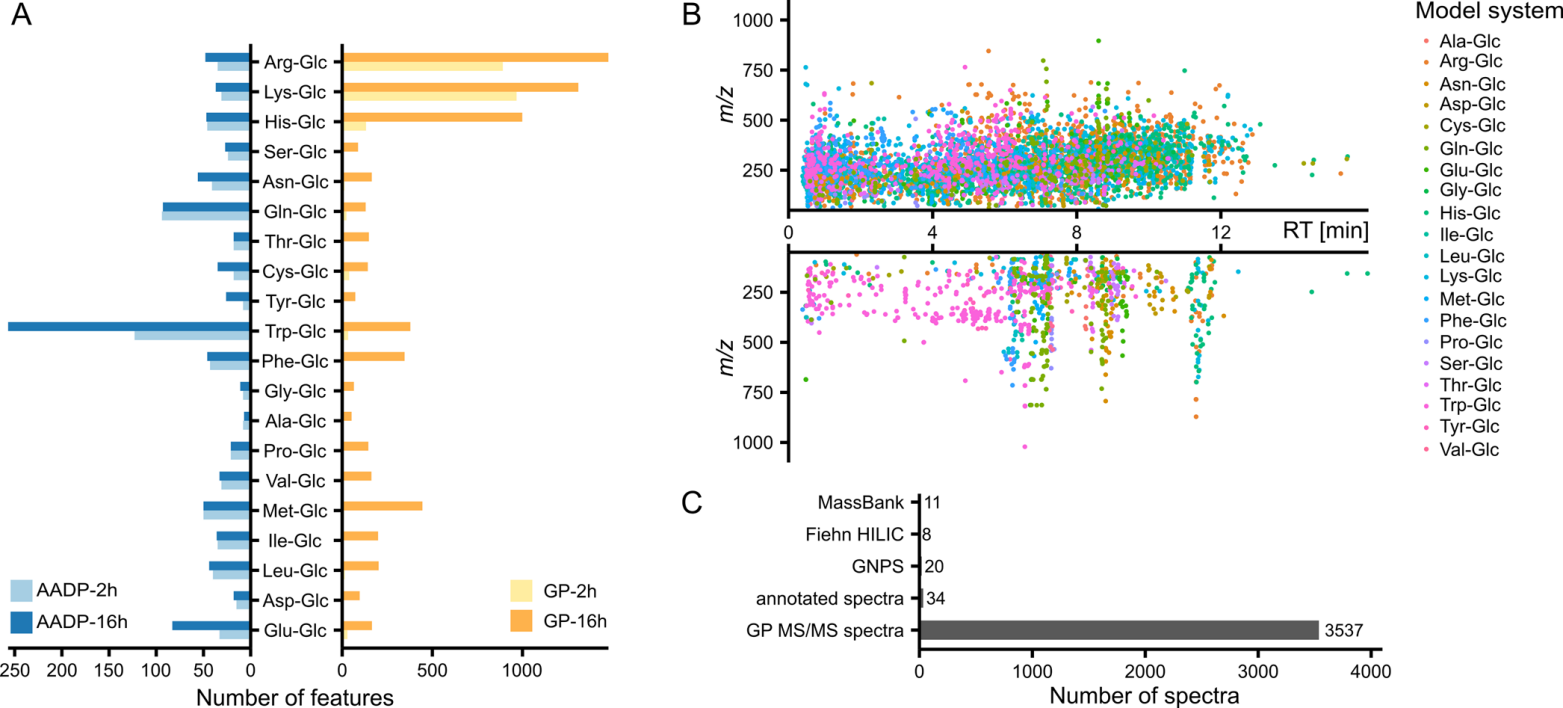
Profiling of GPs in amino acid-glucose model systems. (A) Number of GPs and corresponding AADPs produced in 20 amino acid-glucose model systems heated for 2 and 16 h. Three-letter abbreviations were used for amino acids. Glc: glucose. (B) Distribution of *m*/*z* vs retention time (RT) for GPs (top) and AADPs (bottom). (C) Number of annotated GP MS/MS spectra by matching them with public libraries.

Model systems offer a convenient and reproducible way to generate various GPs, encompassing early ARPs and AGEs. To incorporate GPs with a wide range of chemodiversities, we used 20 proteinogenic AAs to react with Glc separately as model systems. Among all investigated conditions, model systems with basic AAs (Lys, Arg, and His) produced more than twice as many GPs as other model systems (Figure 1A). After 16 h of heating, we can detect the highest number of GP features (1477) in the Arg–Glc model system. Basic AAs tend to have higher reaction rates due to the pH and nucleophilic side chains,^34,35^ which are also mostly subjected to glycation in vivo.^36^ The longer reaction time led to a higher number of GPs, especially for Phe–Glc and Met–Glc, 30 times more GPs formed after 16 h of heating than 2 h. In contrast to the diverse GPs produced in model systems, a smaller amount of CDPs were observed and remained the same after long heat treatment (Figure S1). A similar trend was observed for AADPs in most model systems except for Cys, Tyr, Trp, and Glu, which produced two times more AADPs after 16 h of heating (Figure 1A). The number of AADPs depends on the stability of the corresponding AAs. Trp is known to be the most unstable AA and highly susceptible to light, heat, reactive oxygen species, and so forth induced degradation.^37^ Except for the higher number in model systems, GPs tend to have a larger structural diversity in contrast to AADPs and CDPs. GPs eluted throughout the chromatographic separation range, while AADPs eluted at several distinct elution times (Figure 1B). Among all AAs, Arg, Cys, His, Lys, and Trp produced AADPs with higher structural diversity, which could also contribute to the diverse GPs formation (Figure S2).

Finally, we obtained 7276 GP features with 3537 MS/MS spectra from 20 model systems. We matched the GP spectra with publicly available MS/MS spectral libraries, including GNPS,^25^ MassBank,^26^ and Fiehn HILIC.^27^ Only 34 spectra (0.96%) could be annotated out of 3537 GP spectra (Figure 1C and Table S3), which is much lower than the average annotation rate (∼10%) for small molecules in untargeted metabolomics of biological samples.^27,38^ This suggests that very limited GP spectra exist in public libraries, and most GPs remain so-called dark matter.^39^ For confirming the GP structure, chemical synthesis was conducted in previous studies, which is very time-consuming.^7,8,40^ Considering the large quantity and extensive structure diversity of GPs, it is challenging to establish GP library by targeted synthesis or predict MS/MS spectra based on structural templates. The MR model system is a good source to get experimental MS/MS spectra for GP library creation without reference compounds. The annotation of these unknown GPs from model systems is a challenge that needs to be addressed in the following sections.

### GP Classification and Corresponding Fragmentation Pattern

Despite complex reaction pathways, the MR can be described as the formation and interaction of “chemical pools” resulting from the decomposition of sugar and AAs, a concept presented by Yaylayan.^30^ There are GPs with the same *m*/*z* and RT from distinct model systems annotated as the same compound by a spectral library search, such as pyridoxine (Table S3). It lacks neither the AA backbone nor the specific R-group of AAs in the respective model systems, which explains why it can be detected across multiple model systems. This type of GP could be originated from the interaction between AADPs and CDPs. Besides, *N*-acetyl-AAs, including His and Lys, were detected in the corresponding model systems (Table S3), which could arise from combining intact AAs with CDPs. Building on the conception of “chemical pools” and the structural characteristics of GPs, we classify them into common GPs (CGPs) and MAAs. The structural and fragmentation pattern of each type of GP were first investigated and applied for annotation.

**Figure 2:**
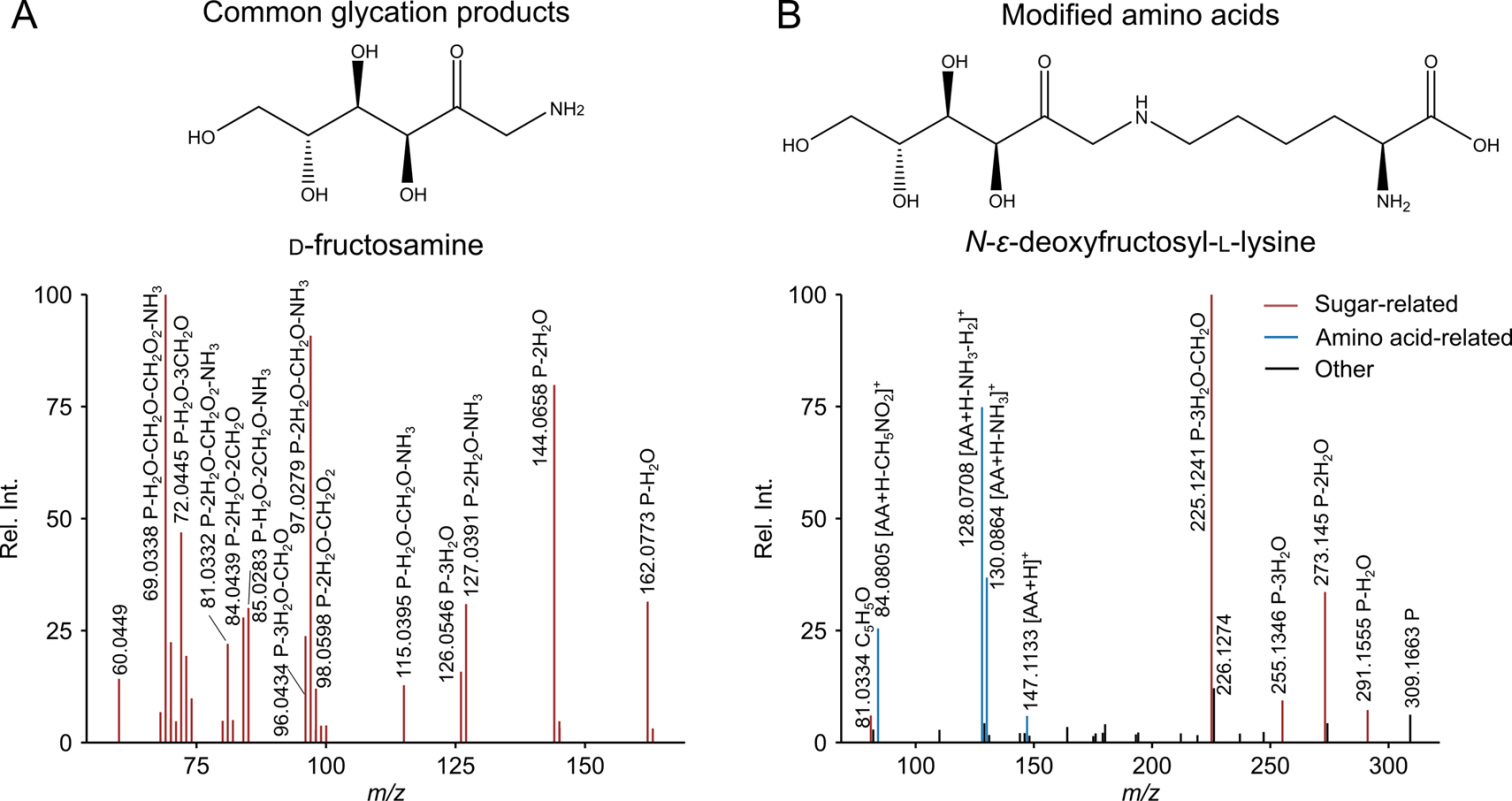
Representative glycation products. (A) Spectrum of a CGP d-fructosamine standard ([M + H]^+^, 180.0866; ion formula, C_6_H_14_NO_5_^+^). (B) Spectrum of *N*-ε-deoxyfructosyl-l-lysine standard ([M + H]^+^, 309.1656; ion formula, C_12_H_25_N_2_O_7_^+^) as an example of MAAs. P represents precursor.

A representative CGP, d-fructosamine, shown in Figure 2A, was detected in 12 out of 20 model systems. Successive water losses at *m*/*z* 162.0761 (C_6_H_12_NO_4_^+^, precursor (P) – H_2_O), *m*/*z* 144.0655 (C_6_H_10_NO_3_^+^, P – 2H_2_O), and *m*/*z* 126.0550 (C_6_H_8_NO_2_^+^, P – 3H_2_O), occur in its MS/MS spectra due to multiple hydroxyl groups in the structure. Its predominant fragments at *m*/*z* 69.0335 (C_4_H_5_O^+^, P – H_2_O – CH_2_O – CH_2_O_2_ – NH_3_), *m*/*z* 72.0444 (C_3_H_6_NO^+^, P – H_2_O – 3CH_2_O), and *m*/*z* 97.0284 (C_5_H_5_O_2_^+^, P – 2H_2_O – CH_2_O – NH_3_) result from bond breaks within the carbohydrate moiety in combination with the loss of H_2_O and NH_3_.^41^ Based on the MR pathway (Figure S3), possible CGPs could include α-aminocarbonyls, heterocycles, and furan derivatives. α-Aminocarbonyls, which contain one nitrogen from AAs and other parts from sugar, can be formed through reductive amination of α-dicarbonyls.^42^ The dimerization of α-aminocarbonyls and reactions with other aldehydes or ketones can form *N*-heterocyclic compounds like pyridines, pyrazines, oxazoles, pyrroles, imidazoles, and so forth.^43^ The cyclization and dehydration of deoxyglucosone via aldol condensation can lead to the formation of furan derivatives.^44,45^ The fragmentation pattern of CGPs varies with the individual types of compounds. Nevertheless, the alignment of feature tables across different model systems aids in the discovery of CGPs, as they can arise from multiple model systems. The lack of corresponding AA-related fragments can further confirm their identity.

In contrast to CGPs, MAAs follow a generic structural template: the amino group of AAs is linked to a modification derived from sugar. Depending on the structure of the modification group, MAAs include AGEs like CML and CEL, as well as amide-AGEs such as glycolic acid-lysine-amide (GALA). Another type of MAAs is cross-links, in which a modification group connects two AAs. One representative MAA, *N*-ε-deoxyfructosyl-l-lysine (FL), is illustrated in Figure 2B. Based on the formation pathway, fragments in its MS/MS can be categorized into AA-related and sugar-related ions. The spectrum exhibits three Lys-related fragments at *m*/*z* 84.0808 (C_5_H_10_N^+^, [AA + H – CH_5_NO_2_]^+^), *m*/*z* 130.0863 (C_6_H_12_NO_2_^+^, [AA + H – NH_3_]^+^), and *m*/*z* 147.1128 (C_6_H_15_N_2_O_2_^+^, [AA + H]^+^). These fragments are also prominent in the MS/MS of Lys and correspond to the loss of formic acid and ammonia from Lys, the loss of ammonia from Lys, and protonated lysine, respectively. In addition, another fragment at *m*/*z* 128.0706 (C_6_H_10_NO_2_^+^), which is not typically found in the MS/MS of Lys but can be generated by the loss of H_2_ from [AA + H – NH_3_]^+^. Neutral losses of H_2_O, 2H_2_O, 3H_2_O, and a combination of 3H_2_O + CH_2_O from fructosyl-moiety also exist in the spectrum, known to serve as characteristic ions for ARPs.^40,46^

By investigating MS/MS spectra of various MAAs, we found that the modification group significantly impacts the fragmentation patterns of MAAs (Figure S4). For the CML, CEL, GALA, and *N*-ε-glycerinyl-l-lysine (GL), their MS/MS spectra closely resemble lysine, with over 90% of the total MS/MS peak intensity explained by AA-related fragments and neutral losses (Table S4). However, for FL as demonstrated above, both AA and modification-related ions contribute to the MS/MS, with only 19% of peak intensity explained by AA fragments. A similar scenario is observed with pyrraline, where only 7% of fragment intensity originated from AAs. Except for FL and pyrraline, more than 50% of peak intensity for other MAAs MS/MS spectra can be attributed to AA-related ions.

Confirmation of whether an intact AA exists in a structure is a requisite for finding an MAA. However, AAs are prone to collision-induced dissociation (CID). Out of the 12 MAAs we investigated, only four produced entire AA fragments as [AA + H]^+^. Notably, three of them are amide-AGEs, including GALA, GL, and glyoxal lysine amide (GOLA), as amide bond is preferential to cleavage by CID.^47^ An alternative way to confirm the presence of complete AAs in MAAs is to identify the pair of [AA + H – NL]^+^ and the corresponding neutral loss (P – NL) in the MS/MS. By combining these two approaches, all 12 MAAs can be recognized. Based on the fragmentation pathway of AAs (Table S5) and investigated MAA standards (Table S4), we summarized fragment-neutral loss pairs for confirming complete AA existence (Table S6).

Furthermore, the modification moiety can be detected either as [modification (Mod) + H]^+^ or [Mod + NH_3_ + H]^+^ ([Mod + 2NH_3_ + H]^+^ for cross-links) depending on whether cleavage of the bond occurs at the C–N connection between the modification group and the AA or at the C–N bond of the AA adjacent to the modification group. Seven out of 12 MAAs exhibit complete modification ions in MS/MS (Table S4). Notably, modifications with a mass less than 50 Da cannot be detected due to *m*/*z* detection range limitations, and modifications susceptible to CID, such as fructosylation, are unlikely to produce complete modification ions.

### Spectral Library Establishment for GPs

**Figure 3:**
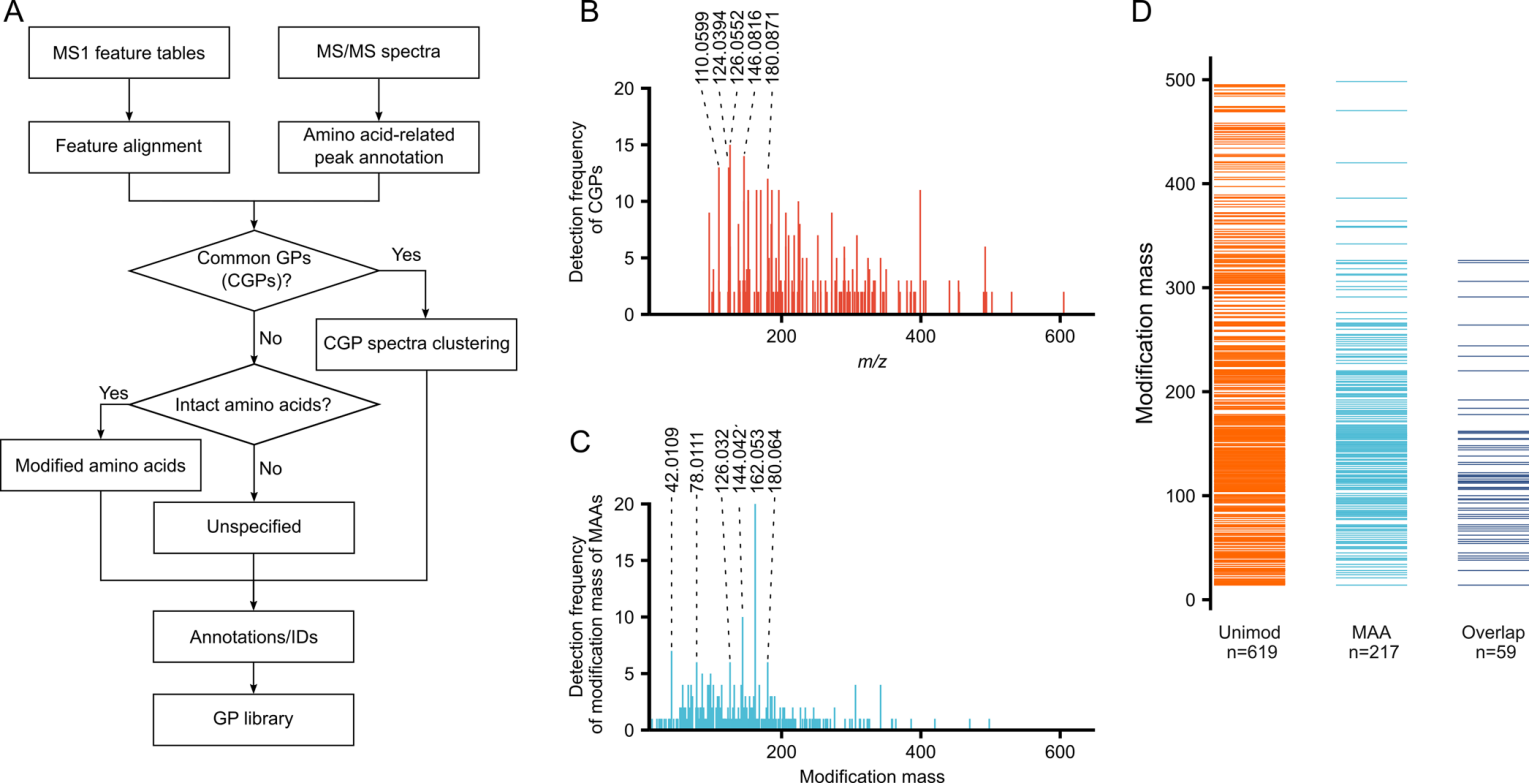
Establishment of GP library. (A) Workflow to annotate GPs. (B) Detection frequency of CGPs across 20 model systems. (C) Detection frequency of MAA modification masses across 20 model systems. (D) Overlap of modification mass between MAAs and protein modifications in the Unimod database.

The workflow for establishing a spectral library for GPs is summarized in Figure 3A. We first aligned the GP features from 20 model systems with an *m*/*z* tolerance of 10 ppm and a retention time tolerance of 0.1 min using feature.align function of apLCMS package.^48^ Pairwise MS/MS cosine similarity for GPs detected in multiple model systems was further calculated. In parallel, MS/MS of all GPs were checked to find AA-related fragments and neutral losses. Based on the detection frequency across model systems and the detectability of AA-related fragments, CGPs were determined by two criteria: (1) the feature can be detected in more than two model systems with an MS/MS cosine score >0.7. (2) Its MS/MS spectra should not contain AA-related fragments in at least one spectrum to remove false-positive CGPs arising from AA isomers (e.g., Leu and Ile). For MS/MS of the same CGP, we retained only the spectrum containing the most fragments in the library and all model system information relevant for CGP generation.

For GPs with AA-related fragments, we confirmed the existence of intact AAs through the detectability of [AA + H]^+^, or pairs of [AA + H – NL]^+^ and corresponding neutral loss (P – NL) in MS/MS. Only compounds encompassing intact AAs meet the criteria to be set as an MAA candidate. The modification mass was calculated by subtracting the *m*/*z* of GPs by the *m*/*z* of corresponding AAs, and then, modification-related fragments [Mod + H]^+^, [Mod + NH_3_ + H]^+^ were checked in spectra. Based on the detectability of modification-correlated fragments, the confidence of MAAs were classified into two levels: level 1, both intact AAs and modification-related fragments were detected. Level 2, only intact AAs were confirmed. In addition, the modification formula was determined by subtracting the AA formula from the formula of MAAs calculated by SIRIUS.^24^ One thing we want to address is that it is hard to determine the structure of the modification group by MS/MS alone, especially for MAAs with small modifications, which have neither modification-related fragments nor neutral losses in MS/MS. Therefore, we provide only the composition of the modification group in our library. The identification in the current study should be considered as “level 3 putatively characterized compound classes”, as defined by the Metabolomics Standards Initiative.^49^

We identified 157 distinct CGPs, comprising 612 spectra and 499 MAAs with 217 unique modifications (Table 1). Most CGPs exhibit *m*/*z* values within the range of 100 to 400, and CGPs with lower *m*/*z* values tend to have a higher detection frequency (Figure 3B). The five most frequently detected CGPs are *m*/*z* 110.0599, 124.0394, 126.0552, 146.0816, and 180.0871, with calculated formulas of C_6_H_7_NO, C_6_H_5_NO_2_, C_6_H_7_NO_2_, C_6_H_11_NO_3_, and C_6_H_13_NO_5_, respectively. These compounds can be linked by mass differences resulting from combinations of dehydration (H_2_O), reduction (H_2_), and oxidation (O), potentially corresponding to amino sugars and derivatives formed through the reductive amination of C6-dicarbonyls with AAs. Furthermore, the compound class predicted by CANOPUS^50^ based on the MS/MS showed that carboxylic acids and derivatives, organooxygen compounds, and pyridines and derivatives are the top three compound classes among CGPs, according to ClassyFire definitions (Figure S5). Pyridine alkaloids, AAs, and amino sugars are the predominant compound classes based on natural product class definitions, which align with the possible CGPs that can be generated based on MR pathways. Compared to MAAs, identical CGPs can be produced by the MR of reducing sugars with distinct AAs, suggesting a higher possibility of being universal markers for MR.

**Table 1:** Annotation Results of GP Spectra

category	spectra	unique ions/modification masses
CGPs	612	157
MAAs	499	217
spectra with amino acid fragments	1142	
spectra without amino acid fragments	1284	

As indicated in Figure 3C, the most shared modifications found across various model systems are 162.053 Da followed by 144.042, 126.032, and 180.064 Da, corresponding to ARP (C_6_H_10_O_5_, 162.0528 Da), its dehydration products ARP – H_2_O (C_6_H_8_O_4_, 144.0423 Da), ARP – 2H_2_O (C_6_H_6_O_3_, 126.0317 Da), and Glc addition (C_6_H_12_O_6_, 180.0634 Da). Similar glycation modifications were observed with high abundance in peptide-Glc model systems.^51^ This aligns with general MR reaction pathway that starts with the formation and degradation of the ARP.^12^ The modification at 42.0109 Da could be attributed to acetylation (C_2_H_2_O, 42.0106 Da). Amide-AGEs, including acetyl-lysine, glycerinyl-lysine, lactoyl-lysine, and formyl-lysine were reported to be stable end products of the MR between lysine and 1-deoxyglucosone through an amine-induced β-dicarbonyl cleavage pathway.^52^ Another modification 78.0111 Da with assigned formula C_5_H_2_O, previously detected in albumins glycated with ribose as well,^53^ could be derived from pyrraline with one CH_2_OH group absent. Typically, free AAs with identical modifications as those found in modified proteins can also be detected in vivo.^54^ To support that observed modifications in our model systems overlap with protein modifications, we compared MAA modifications with those listed in Unimod, which includes a comprehensive collection of natural and artificial modifications.^55^ 59 of these modifications could be matched to known protein modifications from the Unimod database (Figure 3D). Additionally, 158 modifications were found to be unique to our model systems, indicating that the GP library can be helpful for the identification of previously unknown protein glycation modifications.

Some GPs fall outside of these two categories. The library contains 1142 GP MS/MS spectra with AA fragments and 1284 GP spectra without AA fragments. Many of these may be unique to specific model systems such as the interaction of CDPs with AADPs containing AA-specific side chains. Although corresponding annotations are currently not available in the library, we think it is valuable to include these spectra. Matching one spectrum with these can reveal which model system produced the compound. This precursor reactant information can be useful for later structural annotations. To provide comprehensible matching results in library use, GP annotations are integrated in the name following nomenclature: “glycation product type (mz, RT, predicted formula, adduct) detected in specific Maillard reaction model systems”. Additionally, for MAAs, modification masses and explanations from Unimod are included when available. For instance, a MAA entry is named as “modified-tryptophan (*m*/*z*: 247.1083, RT: 406 s, predicted formula: C_13_H_14_N_2_O_3_, adduct: [M + H]^+^) with modification mass 42.0111 [predicted modification composition: C_2_H_2_O_1_, putative annotation from Unimod: acetylation, Unimod composition: H(2) C(2) O] detected in tryptophan-glucose Maillard reaction model system”. Other information, such as confidence levels for MAAs and AA-related fragments detected for unspecified GPs, is incorporated in the comments.

### Identification of GPs by Combination of Spectral Library Search with Simple Model Systems

**Figure 4:**
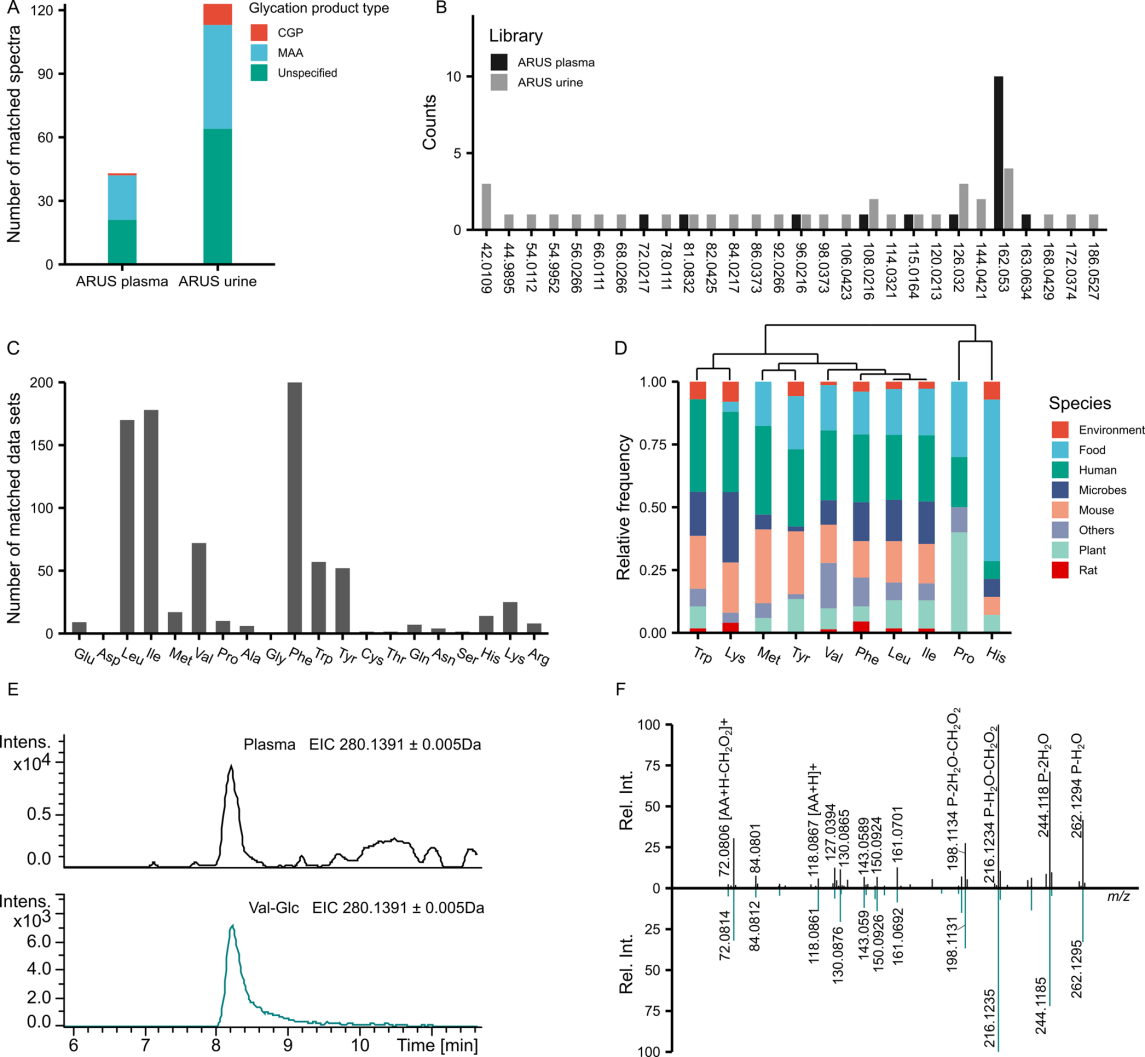
Exploration of free GPs using the established GP library. (A) Number of spectra in GP library with matches in ARUS libraries of human plasma and urine. (B) Distribution of modification masses of detected MAAs in ARUS. Isomers of MAAs derived from the same AAs were counted as one. (C) Number of public metabolomics data sets in which C_6_H_10_O_5_–MAAs were detected using MASST. (D) Distribution of C_6_H_10_O_5_–MAAs found across species. Columns are clustered based on hierarchical cluster analysis (“complete” method). (E) Extracted ion chromatogram of C_6_H_10_O_5_-modified Val in plasma (top) and the Val–Glc model system (bottom). (F) Mirror plot of MS/MS spectra from C_6_H_10_O_5_-modified Val in plasma (top) and from the Val–Glc model system (bottom).

With our library, it is possible to screen potential GPs in untargeted metabolomics studies. To show the existence of GPs in biospecimens as unknowns, we matched our GP library with ARUS from human plasma and urine established by NIST.^14,31^ As shown in Figure 4A, 43 and 123 spectra from our GP library have matches in ARUS plasma (Table S7) and the urine library (Table S8), respectively. This aligns with the prior study showing more GP features were detected in human urine compared to plasma.^17^ The majority of the matched compounds belong to unspecified GPs and MAAs. Upon examining modification masses (Figure 4B), 162.0528 Da (C_6_H_10_O_5_), corresponding to fructosylation/ARPs, present prominently in both plasma and urine. Besides, MAAs matched in urine exhibit a higher diversity of modifications compared to plasma. The level of free AGEs in urine is more prone to be affected by dietary AGEs,^56^ which could be one contribution to the diversity.

To further evaluate the prevalence of GPs in metabolomics data repository, we searched 20 proteinogenic AAs with 162.0528 Da (C_6_H_10_O_5_) modification against all public data in GNPS/MassIVE through MASST.^32^ 18 out of 20 C_6_H_10_O_5_–MAAs, except for Asp and Gly, were present in public data sets (Figure 4C and Table S9). Interestingly, modified Leu, Ile, and Phe were more frequently observed (>100 data sets) rather than basic AAs, which are typically considered to have higher reactivity due to their nucleophilic side chains. To explore the distribution of fructosyl-AAs across species, we checked those detected in over 10 data sets. C_6_H_10_O_5_-modified Trp and Lys were more frequently detected in human samples, whereas C_6_H_10_O_5_-modified Pro and His were observed with a higher frequency in food samples (Figure 4D and Table S10). Free MAAs can originate from the direct glycation of AAs or the degradation of glycated protein. Given the relatively low concentration of free AAs compared to proteins in both living organisms and most foods, variations in the distribution of detected free MAAs are more likely attributed to differences in protein composition across species.

To showcase the utility of the library together with the model system for identifying GPs, we prepared commercial plasma the same way as in the ARUS paper^15^ and analyzed the sample together with model systems. As depicted in Figure 4E,F, C_6_H_10_O_5_-modified Val in plasma and Val–Glc model system exhibited good consistency in RT, peak shape, and MS/MS. Both characteristic sequentially loss of H_2_O, 2H_2_O for ARP, and complete Val fragment at *m/z* 118.0863 (C_5_H_12_NO_2_^+^, [AA + H]^+^) can be detected in the MS/MS. Glycated hemoglobin is a golden criterion for evaluating long-term blood sugar levels in diabetic patients.^57^ The presence of C_6_H_10_O_5_-modified Val could correlate with glycated hemoglobin, as the *N*-terminal Val is the most glycated site on hemoglobin.^58^ The model system can be conveniently and reproducibly prepared. For any matches generated by the GP library, the identity of GPs can be further verified using the model system, which allows for a comprehensive comparison based on three-dimensional characteristics, accurate mass, RT, and MS/MS, without the need for reference standards.

## Conclusions

The low annotation rate of GPs caused by the limited available MS/MS spectra hurdles the investigation of nonenzymatic glycation. Herein, we propose to obtain AA-derived GP spectra by analyzing model systems using an untargeted LC–MS/MS method. Depending on the structural characteristics and fragmentation pattern, we developed a workflow for identifying and annotating two important GPs, CGPs and MAAs. The GP spectral library comprises 157 unique CGP spectra and 499 MAAs with 217 unique modifications. The spectral library can be downloaded as a Mascot generic format (MGF) file from the MassIVE repository with the data set identifier MSV000093499. With our library, potential GPs can be quickly searched for in untargeted metabolomics studies. The output of the library match provides the information on in which model system the matched ions come from and the proposed annotation. More importantly, model systems are straightforward to reproduce and can be used to verify the identity of matches. It also suggests a way for chemically synthesizing the matched compounds and could provide material for orthogonal structural confirmation experiments such as NMR. As more computational spectral annotation tools develop and the community participates, the library will evolve, making GP annotation more comprehensive in the future.

## Supporting Information Available

Number of detected CDPs, distribution of GPs and AADPs for individual model system, MR scheme, MS/MS spectra of MAAs, and compound class of CGPs predicted by CANOPUS (PDF)
Detailed information on standards, LC–MS/MS data processing parameters, library search results for GPs, annotated MS/MS of MAAs, summarized MS/MS of AAs, fragment-neutral loss pairs for confirming complete AAs, matched spectra in GP library with ARUS library from human plasma and urine, and information on matched data sets for C_6_H_10_O_5_–MAAs in the public repository via MASST (XLSX)
