# Supplementary material for: Discovery of Glycation Products: Unraveling the Unknown Glycation Space Using a Mass Spectral Library from In Vitro Model Systems
Source: Anal Chem. 2024 Feb 12;96(8):3569–77. doi: 10.1021/acs.analchem.3c05540 (PMC10902809; doi:10.1021/acs.analchem.3c05540)
Supplement: Supplementary file 1 — ac3c05540_si_001.pdf [file ac3c05540_si_001.pdf]

SUPPORTING INFORMATION

Discovery of Glycation Products: Unraveling the Unknown Glycation Space Using a Mass Spectral Library from In Vitro Model Systems

Yingfei Yan,[†] Daniel Hemmler,[†,‡] and Philippe Schmitt-Kopplin[*,†,‡]

†Research Unit Analytical BioGeoChemistry, Helmholtz Zentrum München, Neuherberg 85764, Germany
‡Chair of Analytical Food Chemistry, Technical University of Munich, Freising 85354, Germany

*Philippe Schmitt-Kopplin (philippe.schmittkopplin@helmholtz-munich.de)

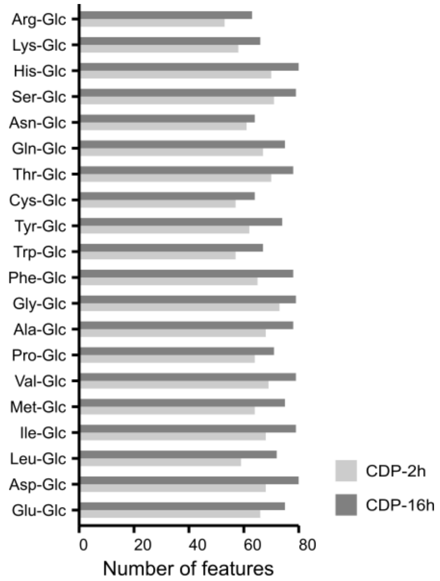

Figure S1. The number of carbohydrate degradation products (CDPs) produced in 20 different amino acid-glucose model systems heated for 2 hours (light grey) and 16 hours (grey).

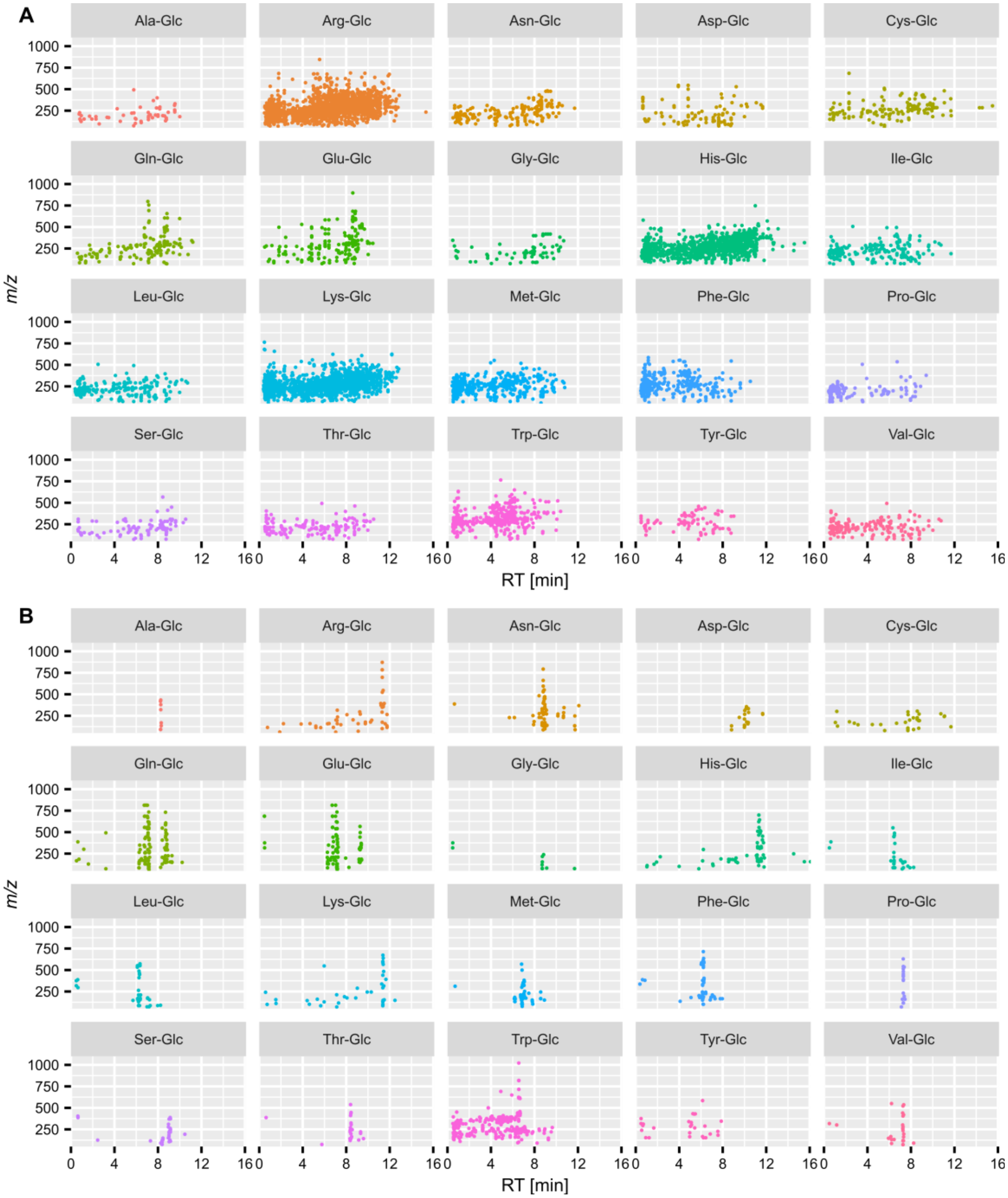

Figure S2. (A) Distribution of *m/z* versus retention time (RT) for glycation products and (B) amino acid degradation products of individual amino acid-glucose (Glc) model system.

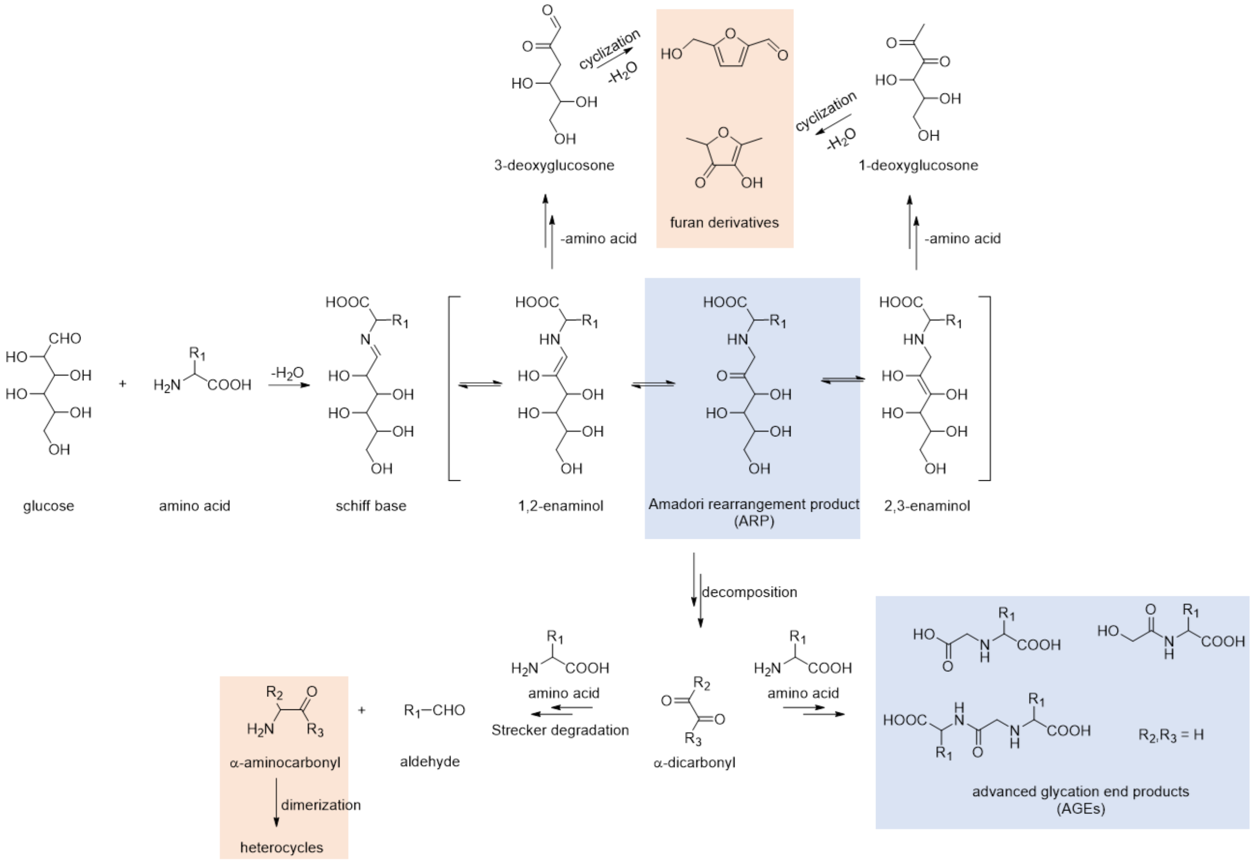

Figure S3. Maillard reaction scheme (Blue: potential modified amino acids; Orange: potential common glycation products).[1–3]

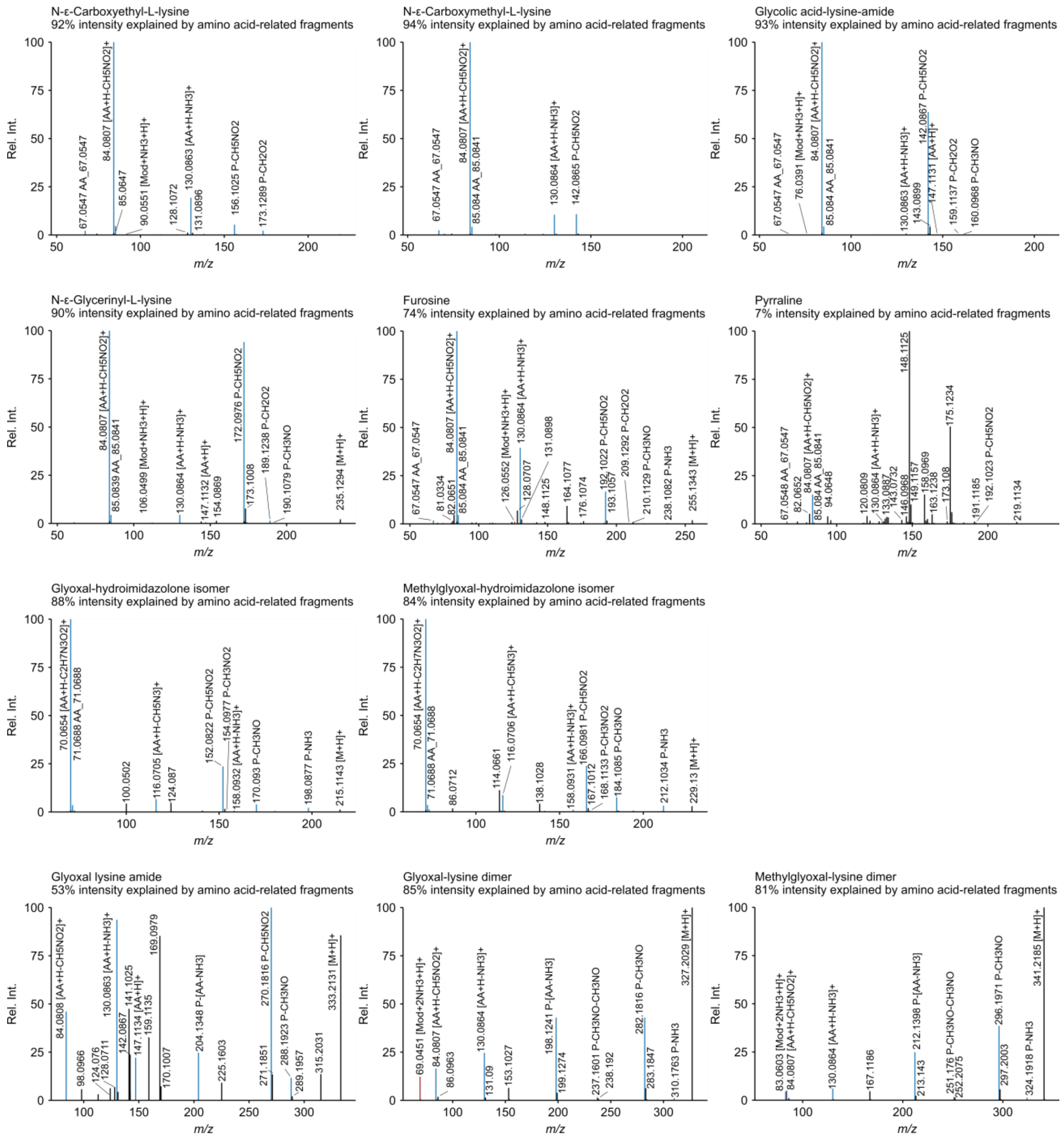

Figure S4. MS/MS spectra of modified amino acid (MAA)-type glycation products with fragments and neutral losses arising from amino acids in blue and modification-related fragments in red.

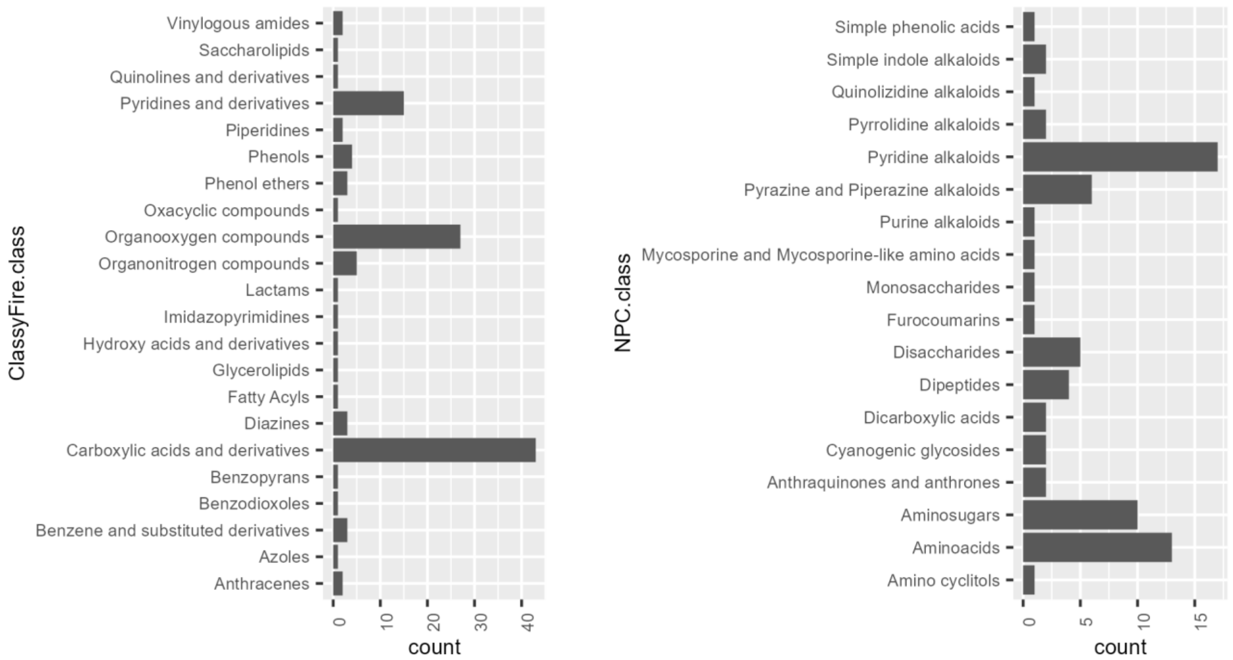

Figure S5. Compound class of common glycation products predicted by CANOPUS with probability > 0.5.[4]

References

(1) Hodge, J. E. Dehydrated Foods, Chemistry of Browning Reactions in Model Systems. *J. Agric. Food Chem.* **1953**, *1* (15), 928–943. DOI: 10.1021/jf60015a004.

(2) Yaylayan, V. A. Recent Advances in the Chemistry of Strecker Degradation and Amadori Rearrangement: Implications to Aroma and Color Formation. *Food Sci. Technol. Res.* **2003**, *9* (1), 1–6. DOI: 10.3136/fstr.9.1.

(3) Lund, M. N.; Ray, C. A. Control of Maillard Reactions in Foods: Strategies and Chemical Mechanisms. *J. Agric. Food Chem.* **2017**, *65* (23), 4537–4552. DOI: 10.1021/acs.jafc.7b00882.

(4) Dührkop, K.; Nothias, L.-F.; Fleischauer, M.; et al. Systematic Classification of Unknown Metabolites Using High-Resolution Fragmentation Mass Spectra. *Nat. Biotechnol.* **2021**, *39* (4), 462–471. DOI: 10.1038/s41587-020-0740-8.
